# Supplementary figures and images for: From caves to seamounts: the hidden diversity of tetractinellid sponges from the Balearic Islands, with the description of eight new species
Source: PeerJ. 2024 Mar 4;12:e16584. doi: 10.7717/peerj.16584 (PMC11636720; doi:10.7717/peerj.16584)

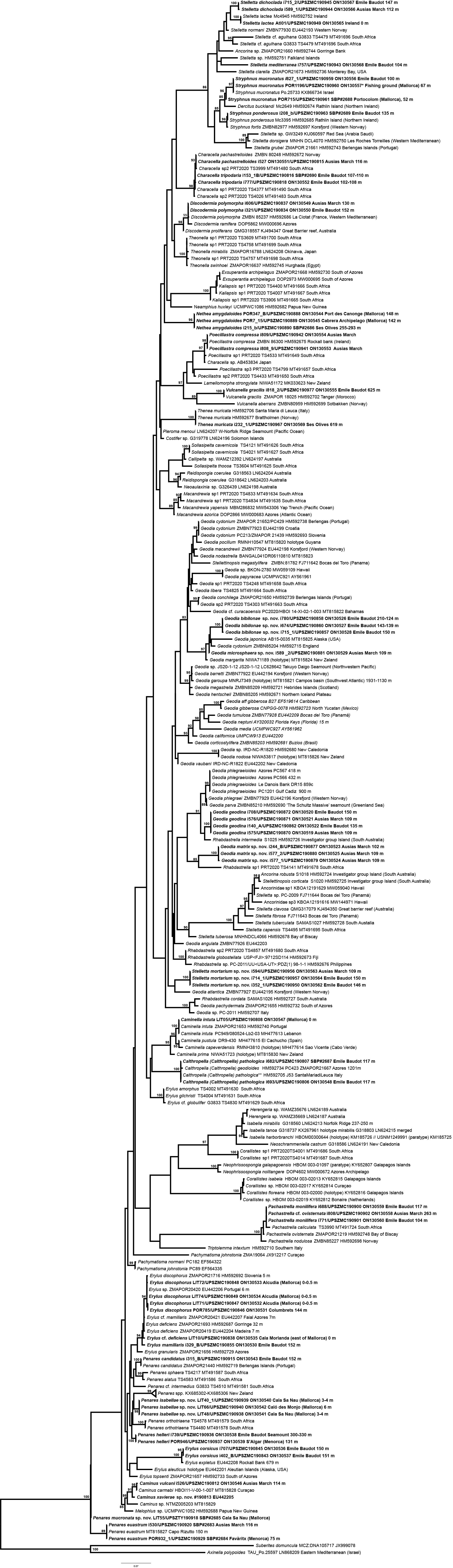

Supplement: Figure S1 — ML bootstrap supports (1,000 bootstrap replicates) ¿80 are indicated. Specimen codes are written as “field number/museum number” followed by Genbank accession number. In bold are new sequences produced in this study. [file peerj-12-16584-s003.jpg]

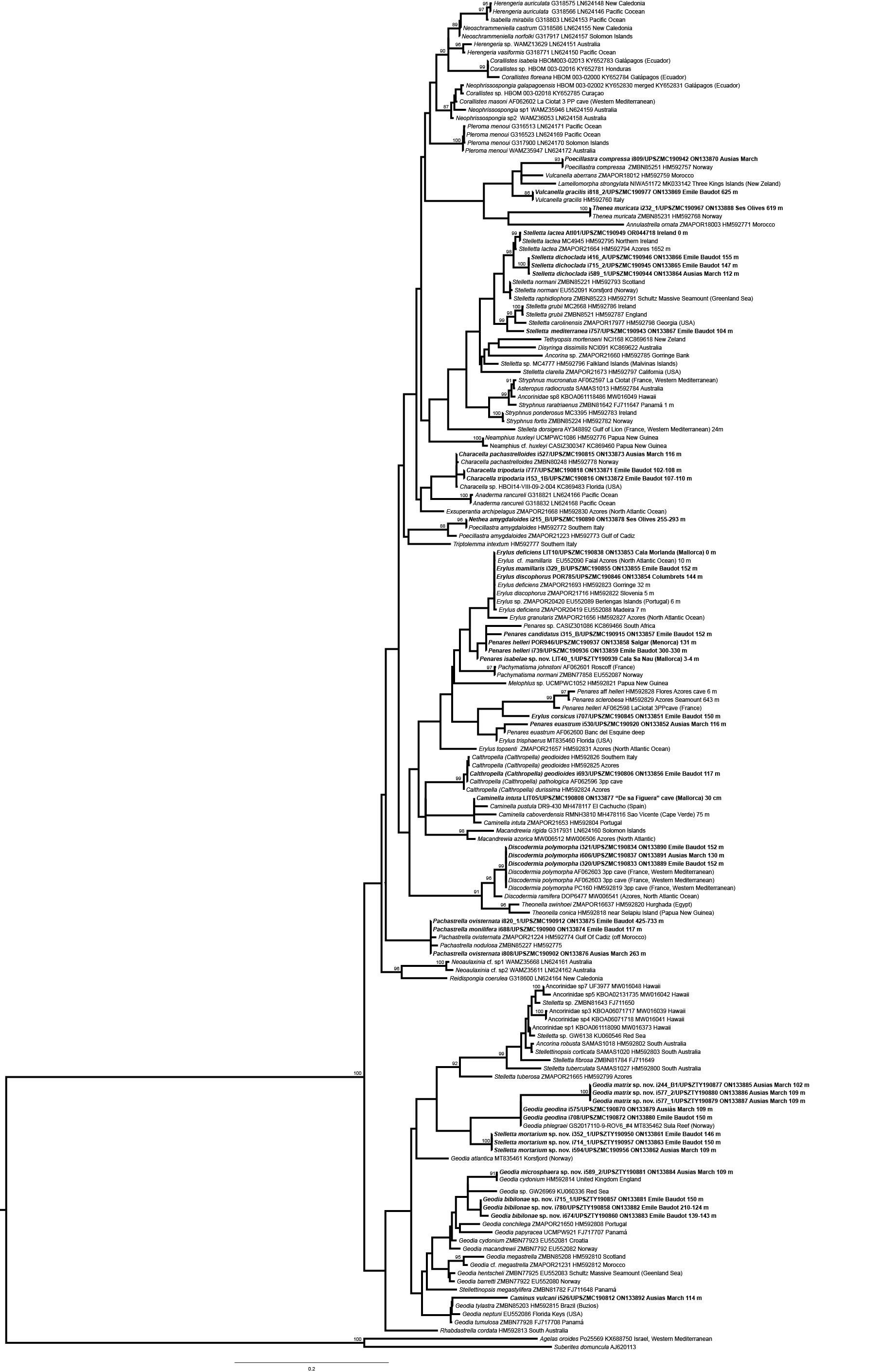

Supplement: Figure S2 — ML bootstrap supports (1,000 bootstrap replicates) ¿80 are indicated. Specimen codes are written as “field number/museum number” followed by Genbank accession number. In bold are new sequences produced in this study. [file peerj-12-16584-s004.jpg]

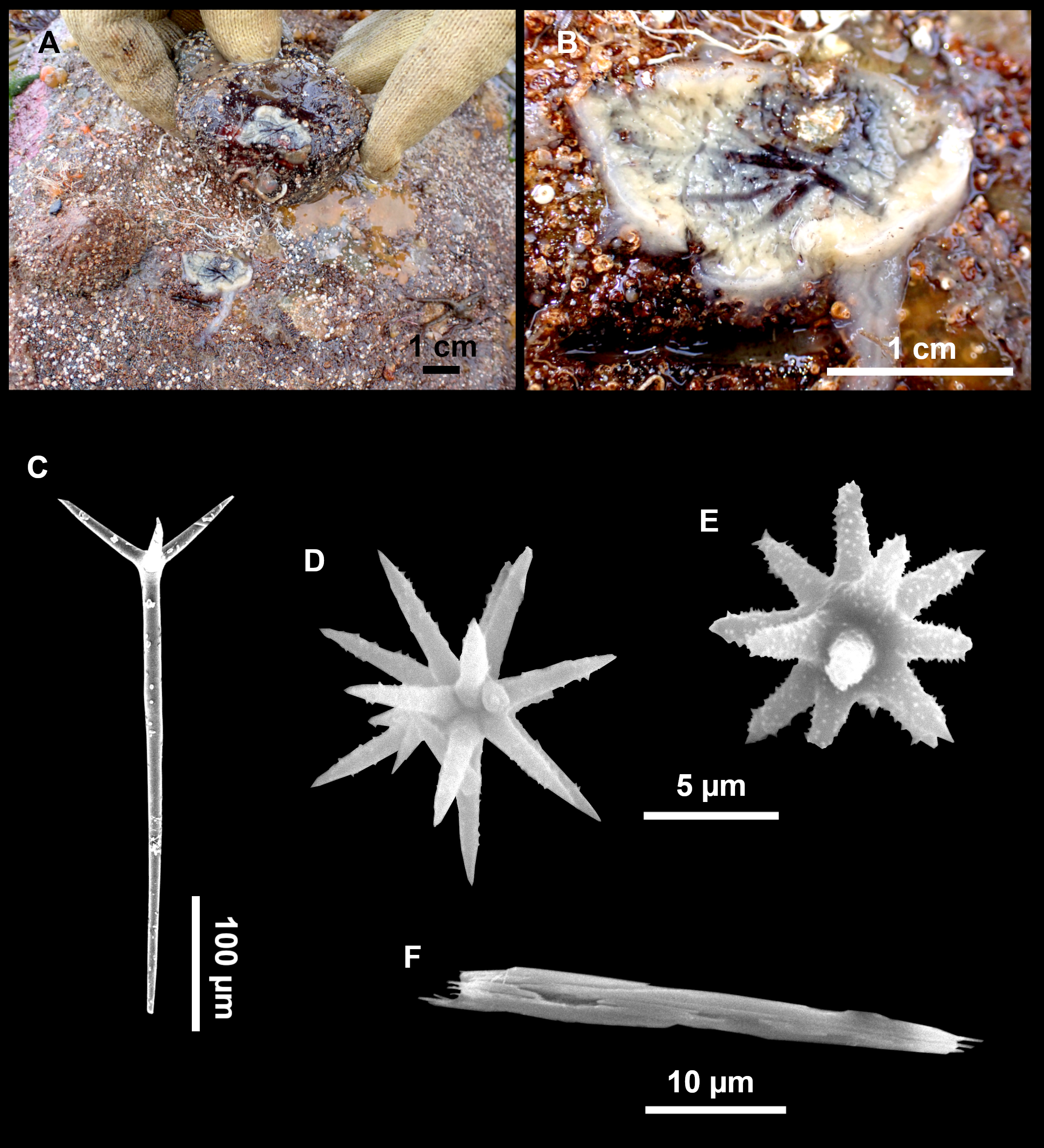

Supplement: Figure S3 — (A–B) In situ images (image courtesy of Christine Morrow). (C) Plagiotriaene. (D–E) Oxyasters to strongylasters. (F) Trichodragmas. [file peerj-12-16584-s005.png]

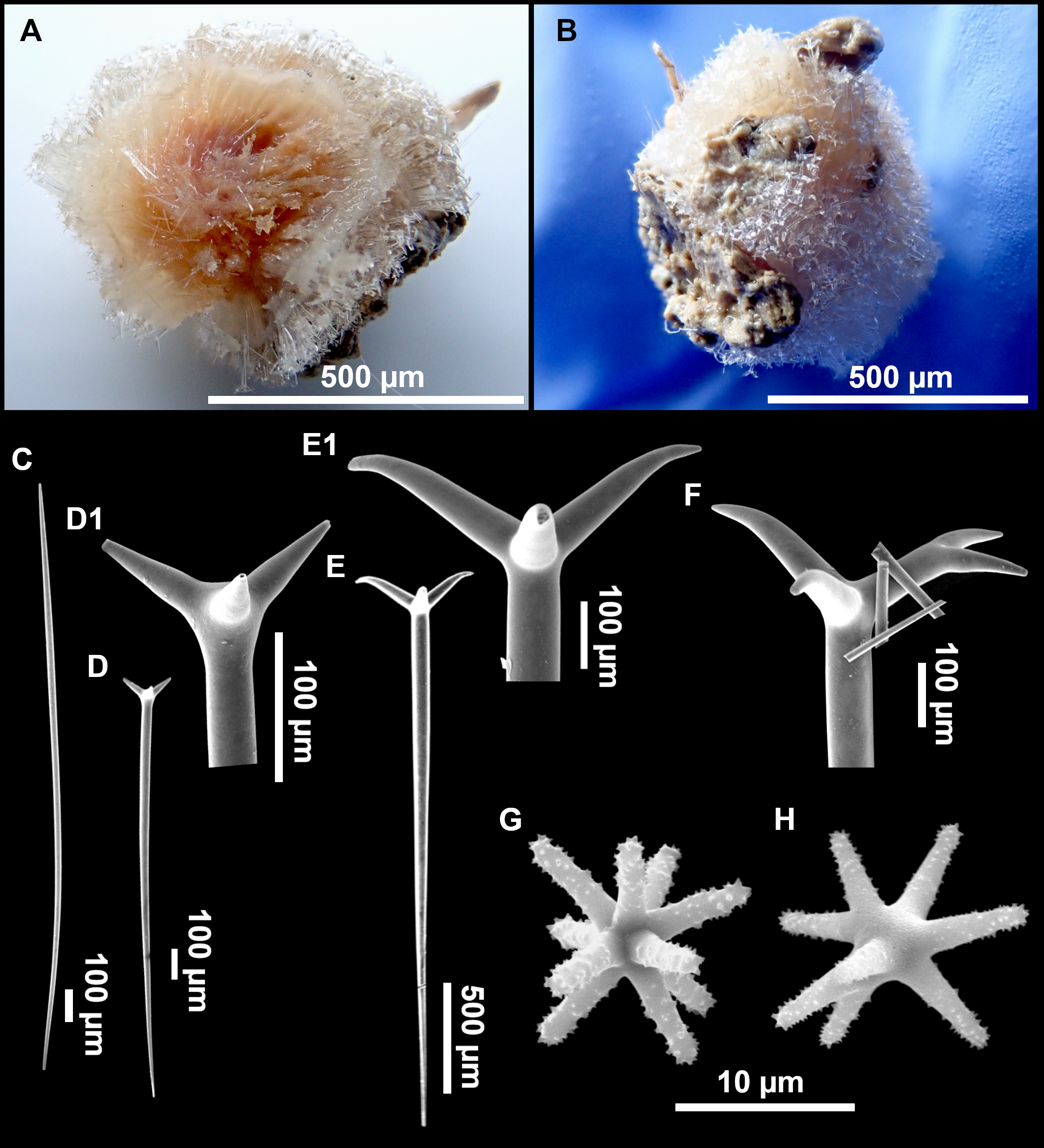

Supplement: Figure S4 — (A-B) Habitus, on a transversal and an upper-body view. (C-H) SEM images of the spicules. (C) Oxeas, (D-D1 and E-E1) Protriaenes with outwards-pointed and curved tips, respectively. (F) Dichotriaene, (G-H) Strongylasters. [file peerj-12-16584-s006.png]
